# Simvastatin Reduces Hepatic Oxidative Stress and Endoplasmic Reticulum Stress in Nonalcoholic Steatohepatitis Experimental Model

**DOI:** 10.1155/2019/3201873

**Published:** 2019-06-18

**Authors:** Graziella Rodrigues, Andrea Janz Moreira, Silvia Bona, Elizângela Schemitt, Cláudio Augusto Marroni, Fábio Cangeri Di Naso, Alexandre Simões Dias, Thienne Rocha Pires, Jaqueline Nascimento Picada, Norma Possa Marroni

**Affiliations:** ^1^Post-Graduation Program in Medical Sciences, Medical School, Federal University of Rio Grande do Sul (UFRGS), 90035-903 Porto Alegre, RS, Brazil; ^2^Research Center, Hospital de Clínicas de Porto Alegre, Federal University of Rio Grande do Sul (UFRGS), 90035-903 Porto Alegre, RS, Brazil; ^3^Post-Graduation Program in Biological Sciences, Physiology, Federal University of Rio Grande do Sul (UFRGS), 90035-903 Porto Alegre, RS, Brazil; ^4^Post-Graduation Program in Hepatology, University of Health Sciences of Porto Alegre (UFCSPA), 90050-170 Porto Alegre, RS, Brazil; ^5^Post-Graduation Program in Pneumological Sciences, Federal University of Rio Grande do Sul (UFRGS), 90035-903 Porto Alegre, RS, Brazil; ^6^Post-Graduation Program in Cell and Molecular Biology Applied to Health Lutheran University of Brazil, 92425-900 Canoas, RS, Brazil

## Abstract

**Objective:**

In this study, we evaluated the efficacy of simvastatin in the treatment of nonalcoholic steatohepatitis induced by methionine and choline-deficient diet in mice and its possible effect on factors involved in the pathogenesis of the disease including oxidative stress and endoplasmic reticulum stress.

**Method:**

Male C57BL6 mice were fed either a normal diet (control) or a methionine and choline-deficient diet for four weeks and then treated orally with simvastatin (4 mg/kg once a day) for two final weeks. At the end of the experimental period, liver integrity, biochemical analysis, hepatic lipids, histology, DNA damage, biomarkers of oxidative stress, and endoplasmic reticulum stress were assessed.

**Results:**

Simvastatin treatment was able to significantly reduce hepatic damage enzymes and hepatic lipids and lower the degree of hepatocellular ballooning, without showing genotoxic effects. Simvastatin caused significant decreases in lipid peroxidation, with some changes in antioxidant enzymes superoxide dismutase and glutathione peroxidase. Simvastatin activates antioxidant enzymes via Nrf2 and inhibits endoplasmic reticulum stress in the liver.

**Conclusions:**

In summary, the results provide evidence that in mice with experimental nonalcoholic steatohepatitis induced by a methionine and choline-deficient diet, the reduction of liver damage by simvastatin is associated with attenuated oxidative and endoplasmic reticulum stress.

## 1. Introduction

The nonalcoholic steatohepatitis (NASH) is a common chronic liver disease, and it has been one of the important factors leading to cirrhosis and hepatocellular carcinoma [1]. NASH is an important stage in the development from simple hepatic steatosis to fibrosis and cirrhosis in nonalcoholic fatty liver disease (NAFLD), characterized by hepatocellular ballooning degeneration and necroinflammation based on hepatic steatosis [2, 3].

The pathogenesis of NASH remains poorly understood. Day and James [4] have proposed the “two-hits” hypothesis for the pathogenesis of NASH based on an animal model that remains a foundation for research in this field. According to this theory, hepatic steatosis is mainly caused by metabolic syndrome (first hit). The second hit includes cellular stresses such as oxidative stress, apoptosis, endoplasmic reticulum (ER) stress, and gut-derived lipopolysaccharide (LPS) [3]. Therefore, the classical “two-hits” hypothesis of progression to NASH is now being modified by a “multiple parallel hits” hypothesis [5]. Among the above factors generating the second hit, oxidative stress is thought to be a major contributor to the pathogenesis and progression of steatosis to NASH [5]. Available treatments are not entirely satisfactory, and taken together, NAFLD and NASH are multidisciplinary liver diseases that require interventions targeting the cardiometabolic and liver disorders for the effective treatment of patients with these diseases [6].

Statins (3-hydroxy-3-methyglutaryl coenzyme A reductase inhibitor) are used worldwide for the treatment of lipid disorders; in particular, they reduce the high levels of low-density lipoprotein cholesterol (LDL-C), decreasing thus cardiovascular events and mortality [7]. However, there are accumulating data in the literature suggesting that statins, such as simvastatin (SIM), may also exert anti-inflammatory effects, such as inhibition of cytokine formation, adhesion molecule expression, and reduction of nitric oxide production [8–11], all of which could be of value in protecting against pathological inflammation and tissue damage.

Trials with larger sample sizes and low risk of bias are necessary before we may suggest statins as an effective treatment for patients with NASH. However, as statins can improve the adverse outcomes of other conditions commonly associated with NASH (for example, hyperlipidaemia, diabetes mellitus, and metabolic syndrome), their use in patients with nonalcoholic steatohepatitis may be justified [12].

In this study, we evaluated the security and efficacy of SIM in the treatment of NASH induced by a methionine and choline-deficient (MCD) diet in mice. The aim of the present study was to evaluate the effects of this drug on disease progression, the liver integrity, and molecular markers of oxidative stress and ER stress.

## 2. Methods

### 2.1. Animals

In this trial, 32 male C57BL/6 mice were used. They were 8 weeks old, weighed 25 grams on average, and were obtained from the Federal University of Pelotas (UFPel), Pelotas, Rio Grande do Sul. The animals were kept in polypropylene cages (47 × 34 × 18 cm), with 4-6 animals in each cage under standard conditions. They were provided with water and food *ad libitum* and maintained on a 12-hour light/dark cycle (light cycle from 7 a.m. to 7 p.m.) under controlled temperature (24 ± 1.0°C) and humidity (55 ± 5%) in the Animal Experimentation Division of the Hospital de Clínicas de Porto Alegre.

### 2.2. Diet Composition

The MCD diet was manufactured by the Brazilian company PragSolucões®, as described by Newberne et al. [13], with modifications as noted in Marcolin et al. [14]. Two types of rat chow were manufactured: MCD and control. The control diet was identical to the MCD diet but contained adequate amounts of methionine and choline.

### 2.3. Experimental Procedure

The experimental protocol complied with the norms established by the Ethical and Health Research Committee of the Group of Research and Postgraduate Studies of the Hospital de Clínicas of Porto Alegre, as well as with the Principles for Research Involving Animals [15]. NASH was induced by feeding the animals with the MCD diet *ad libitum* for 4 weeks. The animals in the control group received the same diet, though with the addition of adequate concentrations of methionine and choline. The animals were randomly divided into four groups (*n* = 8): control+vehicle (CO+V), which received the control diet for 4 weeks plus vehicle; control+SIM 4 mg/kg (SIM 4), which received the control diet for 4 weeks plus simvastatin 4 mg/kg; NASH+vehicle (NASH+V), which received the MCD diet for 4 weeks plus vehicle; and NASH+SIM 4 mg/kg (NASH+SIM 4), which received the MCD diet for 4 weeks plus simvastatin 4 mg/kg. SIM was administered by gavage for 2 weeks. The vehicle was composed of sodium carboxymethylcellulose (CMCNa) 1% and functioned as a carrier for SIM [16].

### 2.4. Experimental Design

On experimental day 1, the animals were randomly assigned to the groups and given their corresponding diets. They were monitored throughout the experiment. Four weeks later, they were weighed and anaesthetized by inhalation of isoflurane so that their blood could be sampled from the retroorbital plexus for liver integrity and biochemical analyses. The animals were killed at 24 h after the last administration of SIM, by exsanguination under deep anesthesia, followed by cervical dislocation as described in the AVMA Guidelines on Euthanasia [17]. The liver was removed by medium ventral laparotomy with total hepatectomy, a part of which was prepared for the histological analysis, while the remaining tissue was frozen in liquid nitrogen for triglyceride and cholesterol concentration, comet assay, oxidative stress, and reticulum stress analysis.

### 2.5. Comet Assay

The alkaline comet assay was carried out as described by Tice et al. [18], with minor modifications. Images of 100 randomly selected cells (50 cells from each of two replicate slides) were analyzed from each animal. Cells were also visually scored according to tail size into five classes ranging from undamaged (0) to maximally damaged [4], resulting in a single DNA damage score for each animal and consequently for each studied group. Therefore, the damage index (DI) can range from 0 (completely undamaged, 100 cells × 0) to 400 (maximum damage, 100 cells × 4).

### 2.6. Liver Integrity Analysis

Liver integrity was assessed by measuring blood levels of enzymes aspartate transaminase (AST) and alanine transaminase (ALT) with standard laboratory methods at Hospital de Clínicas de Porto Alegre.

### 2.7. Total Cholesterol and Triglyceride Analyses

The systemic biochemical analysis included total cholesterol and triglyceride levels. They were measured using standard laboratory methods at Hospital de Clínicas de Porto Alegre.

### 2.8. Hepatic Lipids

In order to measure the hepatic lipid content, frozen liver samples were thawed on ice and homogenized in deionized water. Extraction and isolation of lipids to yield dried lipid extracts were performed. The hepatic cholesterol and triglyceride content of the lipids extracts were then assayed enzymatically by colorimetry.

### 2.9. Histology

For the histological evaluation, a piece of the liver was trimmed and fixed by immersion in 10% buffered formalin for 24 h. The obtained blocks were dehydrated in a graded series of ethanol, embedded in paraffin, and stained with hematoxylin and eosin (HE). The minimum histological criterion for the diagnosis of NASH was the presence of steatosis associated with hepatocellular ballooning involving zone 3 and lobular inflammatory infiltrate. The grading of both necroinflammatory activity and fibrosis was performed according to the classification proposed by Brunt et al. [19].

### 2.10. Lipid Peroxidation, Cytosolic Superoxide Dismutase (SOD), and Glutathione Peroxidase (GPx)

The livers were homogenized with 9 mL of phosphate buffer (KCL 140 mM, phosphate 20 mM, pH 7.4) per gram of tissue. The protein concentration in these liver homogenates was determined using a standard solution of bovine albumin according to Bradford [20]. Liver lipoperoxidation was determined by TBARS [21]. Cytosolic SOD was assayed spectrophotometrically by the rate of epinephrine autooxidation, which is progressively inhibited by increasing amounts of SOD in the homogenate; the amount of enzyme that results in 50% of the maximum inhibition is defined as 1 unit SOD/mg prot. GPx analysis was carried out according to Flohe et al. [22] based on the consumption of NADPH in the reduction of oxidized glutathione, and values were expressed in nmol/mg prot.

### 2.11. Western Blot

Western blot analysis was performed in cytosolic and nuclear extracts prepared from liver homogenates. The supernatant fraction was collected and stored at -80°C in aliquots until use. Protein concentration was measured by the Bradford assay [20]. Lysate proteins were fractionated by sodium dodecyl sulfate-polyacrylamide gel electrophoresis (SDS-PAGE) and transferred to polyvinylidene fluoride (PVDF) membranes. The membranes were then blocked with 5% nonfat dry milk in Tris-buffered saline containing 0.05% Tween 20 (TTBS) for 1 h at room temperature and probed overnight at 4°C with polyclonal anti-NQO1, anti-Keap1, anti-Nrf2, anti-ATF6, and anti BIP/GRP78 antibodies (Santa Cruz Biotechnology, Santa Cruz, CA, USA) at 1 : 200-1 : 1.000 dilution with TTBS in 5% nonfat dry milk. Bound primary antibody was detected (HRP—with anti-mouse IgG, anti-rabbit IgG, or anti-goat IgG antibodies) (Sigma-Aldrich, St. Louis, MO, USA). Protein detection was performed via chemiluminescence using a commercial ECL kit (Amersham Pharmacia Biotech, Little Chalfont, Great Britain) [23]. The density of the specific bands was quantified with imaging densitometer software (Scion Image, Maryland, MA).

### 2.12. Statistical Analysis

The results were expressed as mean values ± SD. The data were analyzed using a Student–Newman–Keuls *post hoc* ANOVA test. The statistical evaluation of data obtained in comet assay was carried out using Tukey's test. Pearson's test (chi-square) was used for correlational analysis. Qualitative variables were subjected to a cross table, and the level of significance was 5% (*P* < 0.05). The software used was SPSS 17.0.

## 3. Results

### 3.1. Comet Assay

There was a significant increase in DI in the NASH+V group (89%) as compared to the CO+V group, suggesting that mice with NASH exhibited increased DNA damage in the liver. The dose of 4 mg/kg SIM was able to reduce the index of DNA damage in animals with experimental NASH. NASH+SIM 4 (-23% DI) was lower than in NASH+V; it was statistically significant. SIM at 4 mg/kg also did not increase DI in comparison to CO+V, suggesting that this dose was not able to induce DNA damage (Table 1)

### 3.2. Liver Integrity

The serum levels of AST and ALT were significantly elevated in the NASH+V group when compared to those in the CO+V and SIM 4 groups, indicating considerable hepatocellular injury. AST and ALT show differences in NASH+SIM 4 when compared with NASH+V (Table 1). Our results demonstrate that a dose of 4 mg/kg has no effect on transaminases in control animals and assists in the reduction of AST and ALT in NASH-induced animals.

### 3.3. Total Cholesterol and Triglyceride Analyses

The serum levels of total cholesterol and triglyceride were significantly reduced in the NASH+V group when compared with those in the CO+V and SIM 4 groups, although no significant differences were observed between the NASH+V and NASH+SIM 4 groups (Table 1).

### 3.4. Hepatic Lipids

Although the difference in serum cholesterol levels of animals with NASH was not observed, when assessing the amount of lipids in the liver tissue, we observed that in the NASH+V group, serum levels were significantly elevated as compared with those in the CO+V and SIM 4 groups (Table 1). However, animals with NASH, treated with SIM 4 mg/kg, demonstrated a decrease (-50%) in the accumulation of cholesterol in the liver compared to NASH animals.

### 3.5. Histology

**Figure 1:**
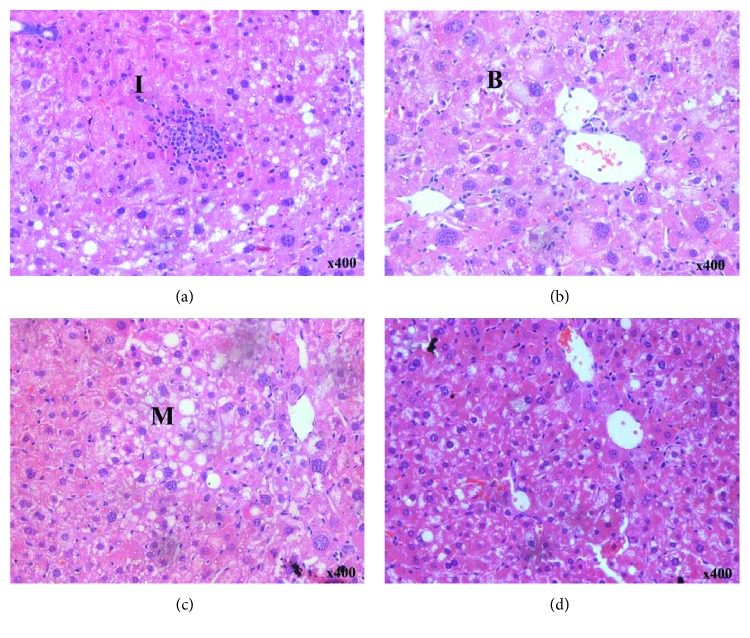
Effect of a MCD diet and treatment with simvastatin. A photomicrograph of the liver of NASH+V (a–c) and NASH+SIM 4 (d) (H-E, 400x). I: inflammation; B: ballooning; M: microvesicular steatosis.

No animal receiving the CO+V diet showed any histological alterations (data not shown). Histological liver changes were observed in animals of the NASH+V group: microvesicular steatosis, macrovesicular steatosis, inflammation, and hepatocellular ballooning (Figures 1(a)–1(c)). SIM administration markedly attenuated the primary ballooning process (Figure 1(d) and Table 2).

### 3.6. Lipid Peroxidation, SOD, and GPx Activity

The analysis of lipoperoxidation (LPO) through TBARS showed a significant increase (300%) in the NASH+V group as compared to that in the CO+V group. NASH+SIM 4 showed a significant decrease (57%) of LPO as compared to NASH+V (Table 3). The analysis of antioxidant enzyme activities showed that SOD activity was decreased in the livers of the NASH+V group (-33%) as compared to those in the CO+V and SIM 4 groups. SOD activity was increased (50.6%) in the treated (NASH+SIM 4) group compared to that in the NASH+V group (Table 3), restoring baseline values. Concerning GPx activity, there was a significant decrease (-64%) in the NASH+V group as compared to that in the CO+V group, and the GPx activity increased (48%) in the treated (NASH+SIM 4) group as compared to that in the NASH+V group (Table 3).

### 3.7. Western Blot

**Figure 2:**
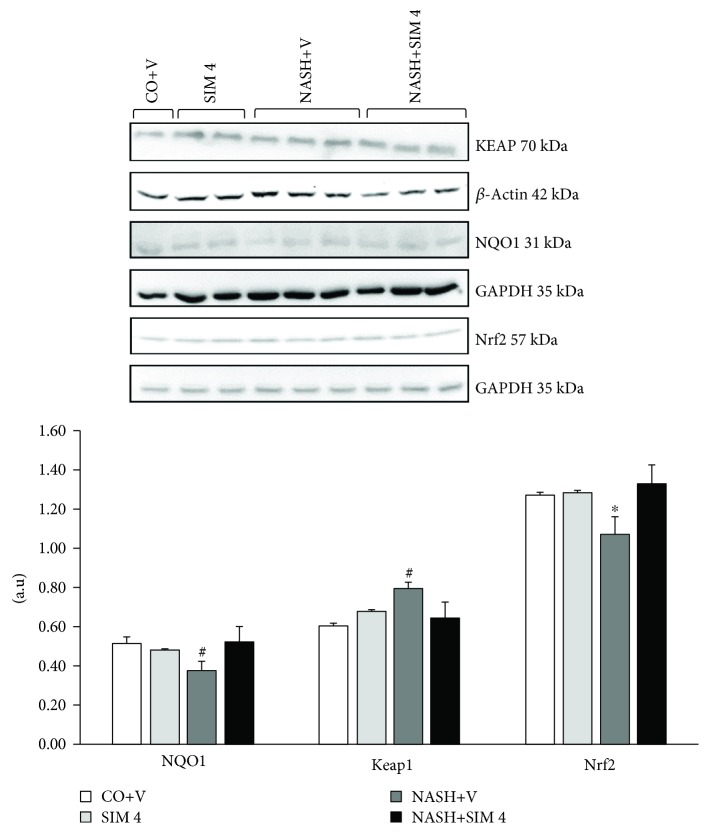
Effect of a MCD diet and treatment with simvastatin on NQO1, Keap1, and Nrf2 protein by Western blot analysis. The data are expressed as the mean ± standard deviation (S.D.). ^#^*P* < 0.01 versus CO+V, SIM 4, and NASH+V. ^∗^*P* < 0.05 versus CO+V, SIM 4, and NASH+V. CO+V: control plus vehicle; SIM 4: control plus simvastatin 4 mg/kg; NASH+V: NASH plus vehicle; NASH+SIM 4: NASH plus simvastatin 4 mg/kg.

**Figure 3:**
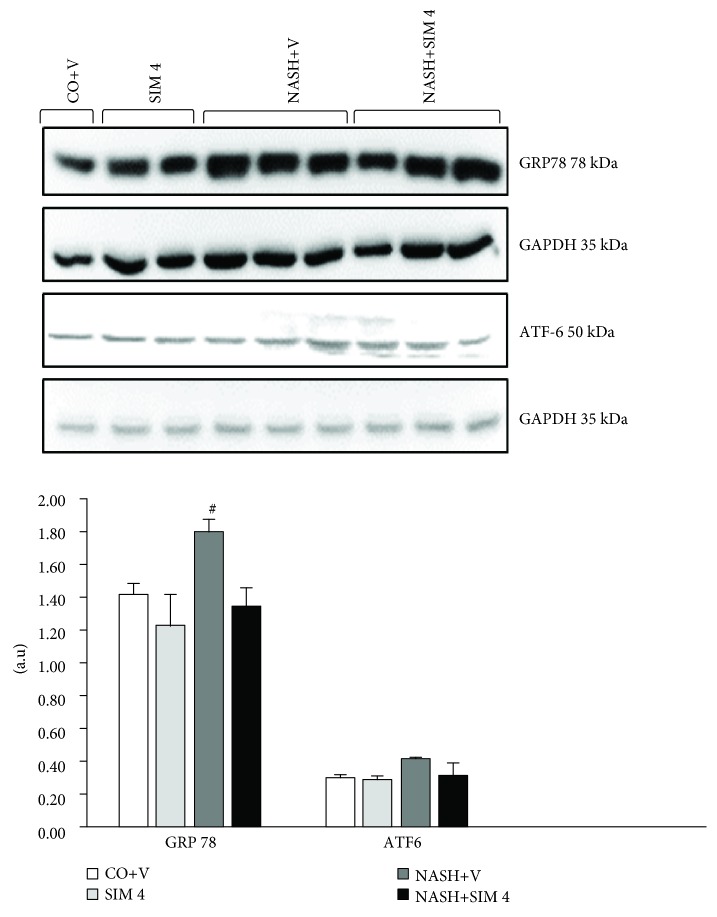
Effect of a MCD diet and treatment with simvastatin on GRP78 and ATF6 protein by Western blot analysis. The data are expressed as the mean ± standard deviation (S.D.). ^#^*P* < 0.01 versus CO+V, SIM 4, and NASH+V. ^∗^*P* < 0.05 versus CO+V, SIM 4, and NASH+V. CO+V: control plus vehicle; SIM 4: control plus simvastatin 4 mg/kg; NASH+V: NASH plus vehicle; NASH+SIM 4: NASH plus simvastatin 4 mg/kg.

Nuclear Nrf2 protein was underexpressed in the NASH+V group. However, the animals in the NASH+ SIM 4 group overexpressed Nrf2. Expression of NQO1 was reduced in animals with NASH, and the NASH+SIM 4 group was able to increase the expression of this protein (Figure 2). Protein markers related to ER stress were also evaluated. The NASH+V group increased the activation of endoplasmic reticulum stress markers, such ATF-6 and GRP78. SIM was sufficient to decrease NASH-induced endoplasmic reticulum stress (Figure 3).

## 4. Discussion

In the present study, we demonstrated that the SIM dose of 4 mg/kg SIM showed favorable results for the attenuation of the disease and does not affect the hepatic integrity, demonstrating safety and no hepatotoxicity. Furthermore, that dose has no hepatic genotoxicity and is shown to be able of reducing oxidative stress and ER stress in C57BL/6 mice with NASH.

The current research has shown that NASH is the most important epidemiological and clinical form of NAFLD. So far, there is no entirely proven treatment for NASH. Statins are an important class of agents to treat dyslipidemia, but there is still reluctance in using this drug in patients with established or suspected chronic liver disease, including NASH. This study sought evidence on the SIM use in liver disease.

The first point was evaluated genotoxicity caused by SIM. We tested three different doses of SIM. In a preliminary study, an increase in DNA damage in the liver of animals that received doses of 7 and 10 mg/kg was observed (data not shown). Our data indicate that there is a relationship between higher doses and toxic effects as evidenced by high rates of DNA damage. A significant increase in DNA damage in the liver was observed in the NASH group, suggesting that this disorder can decrease genomic stability in the liver, corroborating a previous study [14]. Most likely, these effects are implicated in the pathogenesis of NASH, especially concerning the generation of ROS leading to injuries by oxidative stress [24]. Studies have shown that genoprotective or genotoxic effects of SIM are dose-dependent [25]. Here, the 4 mg/kg dosage tested was not able to induce DNA damage evaluated by the comet assay. Animals with NASH showed an increase in DI, and treatment with SIM reduced by 23%, indicating a protective effect on DNA. Furthermore, DI values in the NASH+SIM 4 group were not statistically different from those of the CO+V group, reinforcing that this dosage is not able to induce DNA damage in NASH mice and, conversely, it could be exerting a protective effect.

Recognizing the dose of 4 mg/kg as nongenotoxic, we determined the levels of AST and ALT. They are enzymes that are sensitive to hepatocellular injury. These enzymes in the blood may be associated with centrilobular necrosis, degeneration, and reduced performance state of the liver [26]. In the evaluation of hepatocellular damage, aminotransferase alterations were observed in the animals that received the MCD diet. There are reported cases of statin-related hepatotoxicity, but although elevated aminotransferases are not uncommon in patients receiving statins, serious liver injury from statins is rarely seen in clinical practice [27]. The use of SIM in animals with NASH reduced the levels of these enzymes and reduced the hepatocyte injury.

The MCD diet is a good model for NASH induction, which leads to steatosis due to a decrease in the export of very low-density lipoproteins (VLDLs), such as choline and methionine which are precursors of phospholipid phosphatidylcholine lining VLDL [10]. In the absence of choline, VLDL is not secreted and triacylglycerol (triglyceride) builds up in the liver cytosol. These VLDL fragments are confirmed by our data, which showed a decrease in triglycerides and cholesterol plasma levels along with extensive lipid accumulation in the liver. Accordingly, quantification of the hepatic triglyceride and cholesterol levels showed that MCD diet-induced hepatic lipid accumulation was attenuated by SIM treatment. This decreased hepatic lipid accumulation is extremely important for the outcome of the course of the disease. The reduction of fat on the liver allows ballooning reduction and histological hallmark of NASH and improves liver function, evidenced by the reduction of ALT and AST. In the MCD dietary model of NASH, animals develop steatohepatitis, exhibiting features that are histologically similar to the clinicopathological features of human NASH [14]. Consistent with our expectation, the administration of the MCD diet to C57BL/6 mice resulted in a classical pathophysiological picture of NASH, with microvesicular and macrovesicular steatosis, indicative of disturbed lipid metabolism, multiple foci of inflammatory cell accumulations in the liver, and ballooning.

Statins such as atorvastatin and SIM in clinical studies were associated with a reduction in hepatic steatosis and can inhibit the progression of NASH [28–30], and statins improved indirect markers of liver steatosis, such as serum glyceraldehyde-derived advanced glycation end-products [31]. Treatment with SIM led to a marked decrease in the hepatocellular ballooning degree in the livers of mice fed the MCD diet. Many studies have considered hepatocellular ballooning the main histological feature of NASH and common for the diagnosis of NASH, and it can even be considered a prognostic marker [19, 32].

Oxidative DNA damage is the most common threat to genomic stability. There is strong evidence implicating the generation of ROS and the corresponding responses to oxidative stress as key factors in the pathogenesis of several diseases [14, 33, 34]. Our study showed that SIM decreased damage index to liver DNA. We believe that the protection of the DNA is strongly related to the antioxidant action that the SIM has. Then, we evaluated the oxidative stress and the stress of reticulum in the liver. Our study showed a significant increase in LPO in animals with NASH induced by a four-week MCD diet. This damage can be caused by the increase of ROS in the liver tissue. During NASH, an increased oxidative stress in certain tissues may lead to a rise in the rate of LPO. Similar data were reported in other studies of NASH induced by the MCD diet [14]. SIM administration significantly decreased TBARS levels in the hepatic tissue of animals fed the MCD diet. These results are in agreement with previous studies investigating the effects of SIM [10, 25, 35, 36]. The above result indicates that the SIM can exert antioxidant effects and protect the tissues of lipid peroxidation agents because antioxidant or scavenger of free radicals may be able to inhibit oxidative reactions associated with LPO. Oxidative stress is involved in the development and progression of NASH, and high levels of ROS, due to insufficiency of the antioxidant defense system, may lead to the disruption of cellular function and oxidative damage to membranes, enhancing their susceptibility to lipid peroxidation [4, 5].

Nuclear factor erythroid 2-related factor 2 (Nrf2) is a transcription factor that plays a central role in the antioxidant defense cellular system. It binds to the so-called antioxidant responsive elements (AREs) and regulates the expression of a number of protective genes in response to oxidative stress and electrophiles. Under basal conditions, Nrf2 is retained in the cytoplasm bound to Keap1 which promotes its proteasomal degradation. Upon exposure to ROS or other inducers, Nrf2 is released from Keap1 and translocates to the nucleus where it stimulates the expression of antioxidant genes such as NAD(P)H:quinone oxidoreductase 1 (NQO1) that protect the cell from the deleterious effects of ROS [37–39]. However, Nrf2 expression was greatly reduced in animals with NASH. According to Sugimoto et al. [40], the loss of Nrf2 leads to rapid onset and exacerbation of steatohepatitis in animal models of NASH. Our study showed that SIM activates antioxidant enzymes via Nrf2. Habeos et al. [41] demonstrated that Nrf2, a key regulator of the antioxidant defense, is activated by SIM.

The oxidative stress may be associated with the ER stress. The ER is a cellular organelle essential for cell function and survival. Conditions that interfere with ER function lead to the activation of the unfolded protein response (UPR), a homeostatic signaling network that orchestrates the recovery of ER function, and failure to adapt to ER stress results in apoptosis.

The ER stress response has recently been proposed to play a crucial role in both the development of steatosis and the progression to NASH [42]. The ER stress response is mediated through activating transcription factor 6 (ATF6) and one chaperone, glucose-regulated protein GRP78. In the unstressed state, GRP78 is bound to these transmembrane receptors. However, when unfolded proteins accumulate in the ER, GRP78 preferentially binds to UPR [40].

These markers were induced in animals with NASH due to the use of the MCD diet because this diet is associated with the activation of an ER stress pathway. GRP78 has been recently shown to play a central role modulating the sensitivity and duration of the UPR [43]. The ATF6 pathway also plays a role in stress-induced lipid accumulation [44]. Our study showed that SIM inhibits the ER stress in the liver of the mice with NASH. Although the mechanisms that act to this effect are not fully elucidated, urban et al. [44] showed that the SIM pretreatment exerted a neuroprotective effect by attenuating the ER stress response, with concomitant increases in ATF6 and XBP-1 protein expression during acute ischemia and reperfusion in rats and a novel protective effect of statins against atherosclerosis was proposed based on the finding that steraric acid induced ER stress in macrophages [35, 43, 45].

In summary, simvastatin can help in the treatment of NASH and measures for establishing that meet improvement in cardiovascular and hepatic parameters, besides being safe and cheap drug. Our results support that the reduction of liver damage by SIM treatment is associated with attenuation of oxidative stress and ER stress. However, the molecular mechanisms behind the pleotropic effects are not fully elucidated and further research should be carried out to investigate other molecular mechanisms.

## Figures and Tables

**Table 1:** Effect of treatment with simvastatin on DNA damage, liver integrity, total cholesterol, and triglyceride serum levels and hepatic lipids in mice livers with NASH induced by a MCD diet.

	CO+V	SIM 4	NASH+V	NASH+SIM 4
DI	58.5 ± 15.7	61.68 ± 12.34	111 ± 17 ± 16.97^∗^	85.40 ± 18.82
AST (U/L)	69.14 ± 13.38	74.1 ± 14.50	354.0 ± 62.29^#^	303.7 ± 42.15^#^
ALT (U/L)	30.75 ± 7.52	33.8 ± 7.69	404.0 ± 53.66^#$^	369.0 ± 39.34^#$^
Total Cholesterol (mg/dL)	129.9 ± 13.70	123.1 ± 13.50	28.5 ± 7.73^#^	22.5 ± 4.07^#^
Triglyceride (mg/dL)	67.00 ± 11.88	64.30 ± 8.73	33.6.5 ± 3.70^#^	28.5 ± 2.49^#^
Cholesterol hepatic (mg cholesterol/mg tissue)	0.19 ± 0.03	0.20 ± 0.03	0.40±0.10^∗∗^	0.20 ± 0.02
Triglyceride hepatic (mg triglyceride/mg tissue)	1.54 ± 0.45	1.30 ± 0.42	2.2±0.35^∗∗^	1.7 ± 0.43

CO+V: control plus vehicle (*n* = 8); SIM 4: control plus simvastatin 4 mg/kg (*n* = 8); NASH+V: NASH plus vehicle (*n* = 8); NASH+SIM 4: NASH plus simvastatin 4 mg/kg (*n* = 8). The data are expressed as the mean ± standard deviation (S.D); DI: damage index, which can range from 0 (completely undamaged, 100 cells × 0) to 400 (with maximum damage 100 × 4). ^∗^*P* < 0.05 CO+V versus NASH+V. ^#^*P* < 0.001 versus CO+V and SIM 4. ^$^*P* < 0.05 versus NASH+V. ^∗∗^*P* < 0.05 versus CO+V and SIM 4.

**Table 2:** Effect of treatment with simvastatin on ballooning in mice livers with NASH induced by a MCD diet.

Cross table		Developed the balonization or no		Total
		Without simvastatin	With simvastatin	
Ballooning	None	0	5	5
	Few balloon cells	7	3	10
	Many cell prominent ballooning	1	0	1
Total		8	8	16
		Value	df	Significance
	Pearson's test (chi-square)	11.000^a^	2	.004
	Likelihood ratio	14.230	2	.001
	Linear by linear association	9860	1	.002
		16		16

^a^3 cells (50%) expected a score < 5. The minimum score is 3. Pearson's correlation. *P* < 0.05.

**Table 3:** Effect of treatment with simvastatin on lipid peroxidation, SOD, and Gpx activities in mouse livers with NASH induced by a MCD diet.

	CO+V	SIM 4	NASH+V	NASH+SIM 4
TBARS (nmoles/mg prot)	0.11 ± 0.01	0.1 ± 0.03	0.45 ± 0.12^∗^	0.19 ± 0.02^$^
SOD (U/SOD mg prot)	50.52 ± 12.22	48.9 ± 7.05	33.72 ± 8.74^∗^	50.8 ± 10.92
GPx (nmoles/mg prot)	180.06 ± 57.69	181.59 ± 50.77	114.88 ± 19.46^#^	170.62 ± 34.39

CO+V: control plus vehicle (*n* = 8); SIM 4: control plus simvastatin 4 mg/kg (*n* = 8); NASH+V: NASH plus vehicle (*n* = 8); NASH+SIM 4: NASH plus simvastatin 4 mg/kg (*n* = 8). ^∗^*P* < 0.001 versus CO+V, SIM 4, and NASH+V. ^$^*P* < 0.01 against versus CO+V, SIM 4, and CO+V. ^#^*P* < 0.05 versus CO+V, SIM 4, and NASH+V.

## Data Availability

The data used to support the findings of this study are available from the corresponding author upon request.
